# Immunotherapy for Gastric Cancer: Time for a Personalized Approach?

**DOI:** 10.3390/ijms19061602

**Published:** 2018-05-29

**Authors:** Riccardo Dolcetti, Valli De Re, Vincenzo Canzonieri

**Affiliations:** 1 University of Queensland Diamantina Institute, Translational Research Institute, 37 Kent Str, Woolloongabba, 4102 QLD, Australia; 2 Immunopathology and Tumor Biomarkers Unit/Bio-proteomics Facility, Department of Translational Research and Advanced Tumor Diagnostics CRO National Cancer Institute, 33081 Aviano, Italy; 3 Pathology Department of Translational Research and Advanced Tumor Diagnostics, CRO National Cancer Institute, 33081 Aviano, Italy; vcanzonieri@cro.it

**Keywords:** gastric cancer, immunotherapy, immune checkpoint, chimeric antigen receptor, cancer vaccine, adoptive immunotherapy, Epstein–Barr virus, microsatellite instability, tumor microenvironment

## Abstract

Over the last decade, our understanding of the mechanisms underlying immune modulation has greatly improved, allowing for the development of multiple therapeutic approaches that are revolutionizing the treatment of cancer. Immunotherapy for gastric cancer (GC) is still in the early phases but is rapidly evolving. Recently, multi-platform molecular analyses of GC have proposed a new classification of this heterogeneous group of tumors, highlighting subset-specific features that may more reliably inform therapeutic choices, including the use of new immunotherapeutic drugs. The clinical benefit and improved survival observed in GC patients treated with immunotherapeutic strategies and their combination with conventional therapies highlighted the importance of the immune environment surrounding the tumor. A thorough investigation of the tumor microenvironment and the complex and dynamic interaction between immune cells and tumor cells is a fundamental requirement for the rational design of novel and more effective immunotherapeutic approaches. This review summarizes the pre-clinical and clinical results obtained so far with immunomodulatory and immunotherapeutic treatments for GC and discusses the novel combination strategies that are being investigated to improve the personalization and efficacy of GC immunotherapy.

## 1. Introduction

Gastric carcinoma (GC) is the third most common cause of cancer deaths worldwide with a median overall survival (OS) time for patients diagnosed in a metastatic stage still less than one year [1]. A high proportion of patients diagnosed with GC (≈65%) present with inoperable or metastatic disease, and the survival rate of GC patients decreases dramatically as the tumor stage increases (Table 1). Surgical resection is the primary choice of treatment, with limited resection in stage T1N0; preoperative chemotherapy and surgery, followed by post-operative adjuvant chemo/radiotherapy in stages >T1N0 (advanced tumor); and palliative chemotherapy (supportive care, double, triple regimens ± targeted therapy) in metastatic disease (metastatic tumor) (Table 1). Currently, in non-metastatic advanced GC (>T1N0), the best available systemic therapy combinations only yield a median progression-free survival (PFS) time of 5 to 7 months and a median OS in the range of 8 to 11 months.

Recently, immunotherapy has emerged as one of the most promising strategies in cancer treatment, with outstanding results in several tumor types [3,4,5]. The clinical successes of immune checkpoint inhibitors have revolutionized cancer treatment, clearly indicating that targeting the host’s immune system rather than the tumor may be more effective than conventional therapies. Although encouraging, the results obtained so far in GC patients have, however, still been unsatisfactory, and the majority of novel immunotherapies in this setting are still in the early phases of clinical investigation [6,7]. The most promising response rates obtained so far by this class of immunotherapeutic drugs were induced by pembrolizumab monotherapy, targeting programmed death 1 (PD-1) cells in pre-treated patients with advanced GC [8]. Now, ongoing randomized clinical trials are conducted to assess pembrolizumab’s safety and efficacy in earlier lines of therapy and in combination with chemotherapy for patients with advanced adenocarcinomas of the gastroesophageal junction (GEJ) [9]. Several complex factors are limiting the development of effective immunotherapeutic strategies for GC, including the heterogeneous immunogenicity among and within tumor subtypes and the different and still poorly defined immunosuppressive mechanisms that may hamper effective control of the tumor by host immune cells. In the recently proposed molecular Cancer Genome Atlas (TCGA) GC classification, the *PD-L1* gene was found to be amplified more commonly in Epstein–Barr virus (EBV)-positive and microsatellite instable (MSI)-high GC subtypes with respect to the other subtypes [10,11]. Nonetheless, clinical responses were also observed both in PD-L1- and EBV-negative patients, again highlighting the complexity of the mechanisms underlying the responses to immune checkpoint blockade. Thus, at the clinical level, it is not clear why some patients respond to certain immunotherapies and others do not. Therefore, there are no validated biomarkers allowing reliable discrimination of responders from non-responders. A deeper genetic and immunologic characterization of GC is required to guide patient selection and identify those who could benefit from immune intervention in monotherapy, or more likely, within combination schedules.

## 2. Immunosurveillance and Immunoescape

The critical role of host immunity in controlling cancer development and progression is now well recognized [12]. Data accumulated so far are consistent in indicating that our immune system is able to prevent cancer development through a process termed immune surveillance [12]. This complex process functions through a mechanism of “immunoediting”, which consists of three sequential phases: (1) the elimination phase, in which growing tumors are effectively recognized and cleared by the synergic actions of innate and adaptive immune responses that also recognize remodeling of stroma and changes in the microenvironment; (2) The equilibrium phase, during which, antigen presenting cells, tumor cells and CD8^+^ T cells remain in a state of dynamic balance and the surviving tumor cells remain quiescent under the pressure of immune cells. In this long phase, the immune system of the host sculpts the immunogenicity of genetically unstable tumor clones, allowing for the selection of resistant tumor cells, thus leading to (3) the escape phase, favored by regulatory (Treg) cells and immunosuppressive cytokines, including transforming growth factor-β (TGF-β), Tumor Necrosis factor (TNF)-α, and Interleukin (IL)-10 [12].

Dying cancer cells may express and release tumor-specific and tumor-associated antigens that can be taken up and processed by tissue resident dendritic cells, which then maturate in professional antigen-presenting cells in the presence of an appropriate microenvironment, usually enriched in activator molecules, the so-called danger-associated molecular patterns (DAMPs) [12]. Induction of effective anti-cancer immunity generally requires that mature antigen presenting cells efficiently present tumor antigens in the form of peptides to CD8^+^ T lymphocytes through major histocompatibility complex (MHC) Class I molecules and to CD4^+^ T lymphocytes through MHC Class II molecules. The immunogenicity of tumor antigens varies considerably, the strongest tumor antigens being those provided by non-self or mutated proteins, such as those encoded by viruses or generated by somatic mutations occurring in expressed genes. These latter antigens, the so-called neo-antigens, are generally unique for each individual tumor, thus providing the rationale for personalized immunotherapy. For efficient activation of the CD8^+^ T cells, three different signals are required: T-cell receptor signalling activation after recognition of antigenic peptides in the context of MHC Class I molecules, co-stimulatory molecules, and cytokines provided by professional antigen presenting cells [12]. After activation, T lymphocytes proliferate, infiltrate the tumor, promote the recruitment of other immune cells, and directly kill the cancer cells through the release of cytokines, perforin and granzymes [12]. Incomplete T-cell activation in response to suboptimal amounts of IL-2 or the absence of co-stimulatory signals usually results in T-cell anergy. Another important phenomenon negatively affecting the efficacy of antitumor immune responses is the induction of T-cell exhaustion promoted by the complex network of immunosuppressive cells and cytokines that characterize the tumor microenvironment [13]. T cell exhaustion is a state of altered functionality of these cells, which progressively lose their proliferation, cytokine production, and cytotoxic capabilities. Evidence accumulated so far clearly indicates that exhausted T cells up-regulate the expression of inhibitory receptors, including programmed cell death protein 1 (PD-1), cytotoxic T lymphocyte antigen-4 (CTLA-4), lymphocyte activation gene 3 (LAG-3), T cell immunoglobulin and mucin domain containing-3 (TIM-3), B and T lymphocyte attenuator (BTLA), and T cell immunoreceptor with Ig and ITIM domains (TIGIT) [13,14].

The tumor microenvironment may also impair anti-tumor immunity by promoting the polarization of infiltrating immune cells towards less cytotoxic and pro-inflammatory subsets of T cells (e.g., TH2, TH17 and Treg cells). In GC, the tumor-associated macrophages (TAMs) constitute one of the most abundant immune cell populations present in the tumor microenvironment. These cells can exert anti-tumor activities, or have pro-tumorigenic effects supporting cancer initiation and malignant progression according to differentiation patterns into M1 or M2 subtypes [15]. M1 TAMs exert anti-tumor effects through the release of pro-inflammatory cytokines (IL-1, IL-6, IL-23, TNF-α), whereas M2 TAMs may drive local immune suppression by producing IL-10 and TGF-β. Indeed, TAM infiltration has been shown to functionally inhibit T cells in GC [16,17] and may be a marker of poor prognosis [18,19]. Myeloid-derived suppressor cells (MDSCs) are a heterogeneous population of immature myeloid cells able to inhibit both innate and adaptive immune responses against tumors [20]. These cells are characterized by the ability display have unique features according to the different environments to which they are recruited. The various suppressive properties and functions displayed by MDSCs include increased arginase-1 (Arg-1) and inducible nitric oxide synthase activities, elevated production of nitric oxide and reactive oxygen species, and secretion of various pro-inflammatory cytokines [21]. It has been demonstrated that GC patients have increased numbers of MDSCs in the blood compared with healthy individuals, and this increase was associated with poor clinical outcomes [22]. Another major component of the immune suppressive tumor microenvironment is represented by Treg cells, which may inhibit cytotoxic lymphocytes and/or helper T-cell activity as well as natural killer (NK) cells. Physiologically, Treg cell function is critical to maintain immunological tolerance to self-antigens and suppress excessive immune responses that could potentially be deleterious to the host. Tregs have also been identified as the major regulatory component of the adaptive immune response in *H. pylori*-related inflammation, GC and bacterial persistence [23] as well as in EBV-related GC [24]. A recent study demonstrated that Foxp3^+^CD4^+^ICOS^+^ effector Tregs (eTregs), which has highly suppressive functions, was more abundant in late stage GCs [25]. These tumor infiltrating Tregs exhibited the ability to produce IL-10, but not IFN-γ, TNF-α, or IL-17 and to inhibit the proliferation of responder CD8^+^ T cells.

The presence of tumor infiltrating lymphocytes (TILs) can be detected in various cancers, including GC. Nevertheless, the considerable variability in the number, types and spatial distribution of infiltrates suggests that some tumor types are more immunogenic than others. Indeed, tumors with a low burden of neo-antigens generated by somatic mutations are considered poorly immunogenic and usually show limited or a total absence of infiltration by TILs (immune-desert tumors). The absence of intra-tumoral lymphoid infiltrate may also be due to defects intrinsic to the multi-step T-cell trafficking and homing cascade, a phenomenon that may significantly contribute to immunotherapy resistance [26].

Evidence accumulated so far indicates that TILs may have an important role in influencing the clinical course of various tumors, also including GC [27]. A higher density of both intra-tumoral cytotoxic CD8^+^ TILs and regulatory FoxP3^+^ Treg cells is associated with good prognosis, and this is particularly true for MSI GC, including those that are *H. pylori*- or EBV-positive [24,28]. A recent meta-analysis of 31 observational studies including 4,185 GC patients investigated the significance of the prognostic role of specific T-cell subsets, focusing on overall survival and disease-free survival [29]. In particular, the study concluded that the numbers of CD8^+^, FOXP3^+^, CD3^+^, CD57^+^, CD20^+^, CD45RO^+^, Granzyme B^+^ and T-bet^+^ infiltrating lymphocytes were significantly associated with improved survival (*p* < 0.05). Notably, the amount of CD3^+^ TILs in the intra-tumoral compartment was the most significant prognostic marker (pooled Hazard ratio, HR = 0.52; 95% CI = 0.43–0.63; *p* < 0.001). B-cell activation may also influence tumor prognosis, by producing antibodies against tumor antigens and by activating of a specific B-cell subset (i.e., Breg) that secrete anti-inflammatory mediators (e.g., IL-10) and convert T cells to regulatory T cells (Treg), thus attenuating anti-tumor immune responses [30]. It has been demonstrated that in vivo primed and in vitro activated B cells have showed therapeutic efficacy in adoptive immunotherapy protocols [31,32]. Notably, effector B cells were shown to directly kill tumor cells [32]. On the other hand, resting B cells can promote the development or malignant progression of cancer [33,34].

## 3. Immune-Based Therapies

### 3.1. Adoptive Cell Immunotherapy

The tumor-killing properties of T cells and natural killer (NK) cells provide opportunities to treat cancer. Tumor infiltrating lymphocytes (TILs) and NK cells may have predictive and prognostic relevance in GC [35,36,37,38,39]. Adoptive cell therapies may harness this potential with different modalities. The main strategy involves the isolation of immune cells from a cancer patient, their subsequent genetic modification or treatment to enhance their activity to specifically recognize and kill tumor cells. After adequate ex vivo expansion, these immune cell populations are re-infused into the patient [40]. This process is applicable to most of cancer patients who are unable to mount an effective anti-cancer immunity, and therefore, probably also unable to respond to immune checkpoint inhibitors. There are several different strategies of adoptive cell therapy being used for cancer treatment, most of them have been or are being investigated in the clinical setting for their potential efficacy in GC patients. 

In this setting, MHC Class I-restricted T cells specifically recognizing GC antigens can be successfully isolated from primary tumors, metastatic lymph nodes and ascites from GC patients [41]. However, the limited proportion (about 40%) of biopsies yielding satisfactory T cell populations and the time (about 6 weeks) required to generate adequate numbers of cells for infusion have limited the applicability of approaches using TIL cells [35]. Alternative modalities to generate tumor-specific immune cells have been investigated to overcome these limitations, including the use of cytotoxic T-cell lines generated from the spleen of GC patients [42] or the expansion and re-infusion of T lymphocytes taken directly from a patient’s blood after they have received a cancer vaccine. Indeed, it has been shown that “priming” rare tumor antigen specific T cells first, with active immunization, is associated with more effective expansion of tumor-specific T cells, which can be obtained in greater numbers for therapeutic infusion [35].

The use of in vitro expanded allogeneic NK cells, which have cytotoxic function and the potential to exert antibody-dependent cellular cytotoxicity (ADCC), appears particularly promising for cancer immunotherapy. Compared to autologous NK cells, allogeneic NK cells are more suitable for quality control and large-scale production and have the advantage of not being inhibited by self-histocompatibility antigens, unlike T cells. To expand ex vivo NK cells (over 1000-fold expansion), peripheral blood mononuclear cells of healthy donors or patients are co-cultured in the presence of irradiated K562 leukemia cells that have been modified to express membrane-bound IL-15 and 4-1BB ligands in the presence of IL-2 and IL-15 cytokines in the culture media [43]. However, clinical-grade NK cells at sufficiently high numbers represents a great challenge; therefore, alternative methods to obtain sufficient functional NK cells have been investigated [44,45,46]. Cytotoxic cell lines have been also established from patients with clonal NK-cell lymphoma, and one of them, the NK-92 cell line, has been infused into patients with advanced cancer and showed clinical benefit with minimal side effects [29]. The use of an established NK cell line offers several advantages compared to the use of in vitro expanded NK-cells. Notably, a NK-cell line does not cause graft versus host rejection, and thus can safely be used in allogeneic settings. Based on these considerations, researchers are now exploring the use of engineered NK cells, including the NK-92 cell line, for the treatment of various haematological and non-haematological malignancies. The first chimeric antigen receptor (CAR)-expressing NK-92 cells were generated almost 15 years ago [47]. These cells demonstrated high efficacy against Human Epidermal Growth Factor Receptor 2 (HER2)-positive breast and ovarian cancer cells both in vitro and in vivo [48]. The therapeutic efficacy of this HER2-CAR NK-92 cells has been tested in established mouse models of orthotopic human glioblastoma, renal cell and breast carcinoma [49]. Results of these studies demonstrated specific homing of the NK cells to the tumor sites, a reduction in the number of metastases and significant tumor regression, indicating that this could constitute a promising therapeutic approach for HER2+ GC.

Another adoptive cell therapy approach is based on the exploitation of the immunotherapeutic properties of a heterogeneous population of immune effector cells: the cytokine-induced killer cells (CIK). These cells can be obtained by treating peripheral blood lymphocytes with interferon-γ (IFN-γ), a monoclonal antibody against CD3 and an interleukin (IL)-2 [50]. CIK cells are mainly expansions of CD3^+^CD8^+^CD56^−^ negative cells to terminally differentiated CD56-positive natural killer (NK) T cells. These cells have the peculiar capacity of recognising tumor cells both in the presence and in the absence of antibodies and MHC; thus, they can also recognise tumor cells that are missing MHC molecules on their surfaces. The cytotoxicity of CIKs is mediated by perforin release and is dependent on the interaction between killer cell lectin like receptor K1 (NKG2D) and NKG2D ligands. Moreover, in vivo CIK cells can also regulate and increase host cellular immune function through the secretion of several cytokines and chemokines. Available evidence indicates that combination therapy with chemotherapy and CIK generally improves the progression-free survival (PFS) and overall survival (OS) times of patients with cancer, including GC (Table 2). Some chemotherapies (e.g., doxorubicin, mitoxantrone, oxaliplatin and cyclophosphamide) may add positive immune effects by fostering CD8^+^ T-cell infiltration into the tumor and promoting the release of tumor antigens through the induction of immunogenic death of tumor cells [51]. Two meta-analyses considering relevant clinical trials concluded that CIK cell therapy significantly increases the 5-year OS rate of GC patients compared to conventional chemotherapy, thus providing statistical evidence to support the activation of large-scale clinical trials with CIK cell therapy [52,53]. Interestingly, the percentage of lymphocyte subsets (CD3^+^, CD4^+^ and CD3^−^CD56^+^, CD3^+^CD56^+^; *p* < 0.01) and the levels of IL-12 and IFN-γ, which reflect immune function, were significantly increased (*p* < 0.05) after the CIK/DC-CIK therapy [53]. A particularly attractive perspective for the clinical exploitation of CIK cells is their combination with monoclonal antibodies [54]. Indeed, pre-clinical evidence has been provided indicating that CIK cells combined with a monoclonal antibody against epidermal growth factor receptor (EGFR) enhance the antitumor ability of CIK cells both in vitro and in vivo [55]. 

In summary, the overall data reported so far indicates that autologous immune cell administration with adjuvant chemotherapy is associated with better prognosis for patients with GC compared to those treated with chemotherapy only [34]. Some examples are reported in Table 2. Nevertheless, current approaches of adoptive cell-based immunotherapy need to be improved to make clinical application more feasible. In this respect, it has been shown that T/NK cell-mediated anti-tumor activity may be suppressed by tumor or stromal cells via inhibitory soluble factors/cytokines or through the engagement of inhibitory immune checkpoint molecules. These findings strongly suggested that blocking inhibitory regulators of T/NK cells might be an attractive and promising strategy to increase the efficacy of T/NK cell-based tumor immunotherapy [56].

### 3.2. Engineered Cells for Adoptive Immunotherapy

**Figure 1:**
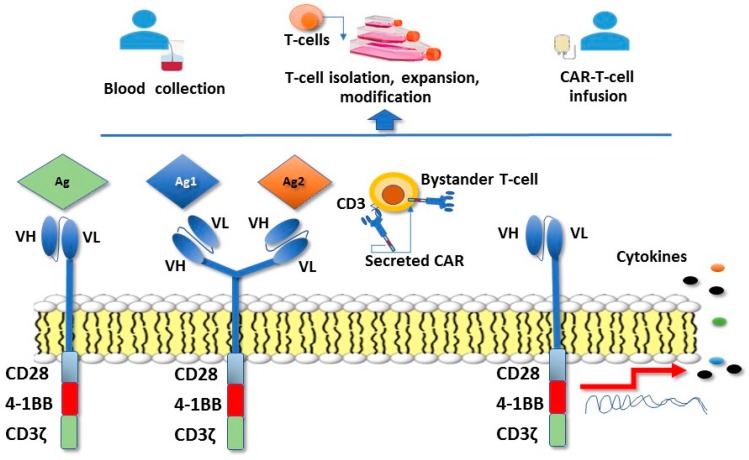
Chimeric antigen receptor (CAR)-T cell therapy T cells are isolated from blood of the patient or a donor, activated, and genetically engineered to express the CAR construct. Engineered CAR-T cells are then reinfused into the patient. The extracellular portion of CAR-T cells is a ligand-binding domain composed of a B cell receptor-derived single-chain variable fragment (VH-VL), whereas the T-cell receptor molecule signalling domain is composed of CD3 molecules and a ζ-chain (zeta chain) and one or more intracellular costimulatory domains required for T-cell stimulation (i.e., CD28 and 4-1BB or CD137). CAR-T cells can also be engineered to recognize two different antigens (dual specificity CAR-T cells). In addition to classical CAR-T cells, new CAR T-like constructs are also able to secrete soluble forms of the CAR receptor. The secreted CAR construct was demonstrated to be able not only to directly kill HER2+ GC, but also to transfer this ability to bystander T cells. More recent approaches have been based on the use of CAR-T cells genetically modified to express CARs along with a gene cassette driving the expression of cytokines (red arrow) that enhance T-cell activity.

To broaden the applicability and enhance the efficacy of adoptive cell therapy that could potentially lead to the elimination of the tumor cells, techniques have been recently developed to introduce antitumor antigen receptors into normal T cells that could be then used for therapy. The specificity of T cells can be redirected towards tumor cells by the use of viral vectors, allowing the expression of CARs specific for tumor antigens [64,65]. The T-cell receptor (TCR) recognition process requires antigen presentation via the major histocompatibility (MHC) complex. However, a significant proportion of tumors down-regulate MHC expression to escape immune surveillance. Engineering T lymphocytes with chimeric antigen receptors (CAR) and combining B cell receptor-derived and T cell receptor domains, has the advantage of bypassing the need for MHC interaction and costimulatory molecules. The extracellular portion of CAR-T cells is a ligand-binding domain composed of a B cell receptor-derived single-chain variable fragment, whereas the signalling domain is composed of CD3ζ and one or more intracellular costimulatory domains (Figure 1).

The adoptive transfer of CAR-T cells has so far demonstrated promising antitumor effects in advanced hematologic malignancies, but only limited benefits in patients with solid tumors. This may be due to the heterogeneous tumor antigen expression, immunosuppressive networks in the tumor microenvironment, the suboptimal trafficking of T cells into solid tumors and the lack of effective costimulatory signals required for CAR-T persistence after infusion [64,65,66]. In pre-clinical models of GC, treatment with CAR-T cells specific for the HER2 oncoprotein as well as the use of a bifunctional αHER2(Ag1)/CD3 (Ag2) RNA-engineered CAR-T-like human T cells, induced a marked regression of the tumor and prolonged the survival of tumor-bearing mice [67,68]. Of note, in addition to classical CAR-T cells, CAR T-like constructs also able to secrete soluble forms of the CAR receptor were able not only to directly kill HER2+ GC, but also to transfer this ability to bystander T cells [68]. Another HER2-targeting CAR-T constructs harboring T-costimulatory molecules (i.e., 4-1BB, CD3ζ exhibited a considerably enhanced tumor inhibition ability and was able to promote long-term survival and T-cell homing to GC xenotransplanted mice [69]. CAR-T cells were also shown to eliminate patient-derived GC stem-like cells, an important effect to search for and implement, to enhance the possibility of eradicating tumor cells [50]. A phase I/II clinical study (NCT02713984) involving patients with several HER2-expressing tumor types, including GC, and treatment with HER2-targeting CAR-T cells is ongoing. Another therapeutic target antigen for GC is the Human Carcinoembryonic Antigen (CEA), an oncofetal glycoprotein overexpressed in gastrointestinal carcinomas. With the aim of enhancing the antitumor activity and in vivo persistence of CAR-T cells, CAR-T were engineered with a construct, combining CEA with a fusion protein of IL-2. In comparison with free IL-2, the combination of CAR-T cells with IL-2 significantly enhanced the antitumor activity against human GC cell line MKN-45 cells [70]. Several phase I studies are investigating the safety and therapeutic efficacy of CAR T cells redirected towards different GC antigenic targets, including CEA, MUC1 (mucins lining the apical surface of epithelial cells in GC) and EpCAM (an epithelial cell adhesion/activating molecule) (Table 3).

Despite the efficacy shown by CAR-T-cell therapy in some clinical settings, this novel treatment strategy may be burdened by unique acute toxicities, which can be severe or even fatal [71]. Cytokine-release syndrome (CRS) is the most frequently observed adverse event, which can range in severity from low-grade constitutional symptoms to a high-grade syndrome associated with life-threatening multi-organ dysfunction. Only rarely, severe CRS can evolve into fulminant haemophagocytic lymphohistiocytosis. Neurotoxicity, defined as CAR-T-cell-related encephalopathy syndrome, is the second most frequent adverse event, and can occur concurrently with or after CRS. Considering that antigens on cancer cells may be also expressed on normal cells, on target off-tumor toxicity can occur upon stimulation of T cells following the binding of CARs to their antigens on the normal cells/tissues. Life-threatening on target off-tumor toxicity may particularly occur in cases in which the target antigen is expressed in vital tissues such as the respiratory system. This fatal occurrence was reported in a patient with metastatic colorectal cancer following the administration of ERBB2 CAR-Ts where low expression of ERBB2 on respiratory normal epithelial cells led to acute pulmonary manifestation and the patient’s death 5 days after the injection of CAR-Ts [72]. New strategies such as designing CAR-Ts with limited life-span or “on-switch CARs” are under investigation to ameliorate the toxicity of CAR-T.

### 3.3. Immune Checkpoint Inhibitors/Immune Modulatory Pathways

**Figure 2:**
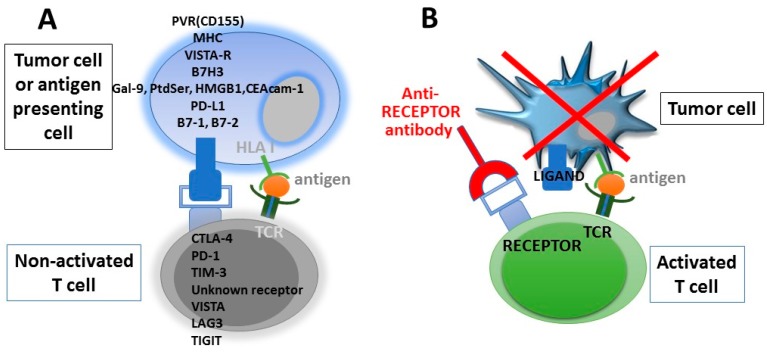
Blocking the immune checkpoint restores the ability of tumor-specific T lymphocytes to kill tumor cells. Antibodies/agents against receptors on T cells (i.e., CTLA-4, PD-1, etc.), and/or their relative ligands (i.e., B7, PDL-1, etc.) on antigen presenting cells or tumor cells re-activate pre-existing anti-tumor T cells that can induce tumor cell killing. Recognition of the human leukocyte antigen (HLA) Class I/peptide antigen complex by the T-cell receptor present on T cells is required to induce tumor cell killing; (**A**) Inhibitory receptor/ligand interaction is not blocked and the tumor cell is not killed; (**B**) the immune checkpoint receptor is blocked by an inhibitory antibody and the T-cell is re-activated and is thus able to kill tumor cells. PVR: poliovirus Receptor; MHC: Major Histocompatibility Complex; VISTA: V-domain Ig suppressor of T cell activation; VISTA-R: VISTA Receptor; Gal-9: Galectin-9; PtdSer: Phosphatidylserine; HMGB1: High Mobility Group Box 1; CEAcam-1: Carcinoembryonic antigen-related cell adhesion molecule 1; PD-L1: Programmed death-ligand 1; CTLA-4: Cytotoxic T-Lymphocyte Antigen 4; PD-1: PD-L1: Programmed death 1; TIM-3: T cell immunoglobulin and mucin domain 3; LAG-3: Lymphocyte-activation protein 3; TIGIT: T-cell immunoreceptor with Ig and ITIM domains.

Immune checkpoint therapy exploits the function of molecules that physiologically regulate and balance immune responses by inhibiting T-cell activation or, alternatively, by activating stimulatory pathways with the final result to maintain homeostasis and avoid tissue damages due to excessive immune activation. In the field of cancer immunotherapy, these treatments are designed to release or enhance pre-existing anti-cancer immune responses. Indeed, tumor cells may induce T-cell suppressive signalling to successfully evade immune-mediated tumour eradication, a phenomenon called adaptive immune resistance. The inhibitory signals suppressing T-cell activation are mediated by a variety of “immune-checkpoint” molecules (inhibitory ligands and their cognate receptors), including the CD28/cytotoxic T-lymphocyte antigen 4 (CTLA-4) axis, and PD-L1/PD-1 pathway, which have emerged as promising targets. Other checkpoint molecules, such as TIM3, B7H3, VISTA, LAG3, and TIGIT, are currently being evaluated as potential targets for cancer immunotherapy [73] (Figure 2). Pathways involving these regulatory molecules are crucial for maintaining the tolerance against self-antigens and modulating the duration and amplitude of immune responses against non-self or mutated tumor antigens in order to avoid tissue damage. When these negative regulatory proteins are blocked, the inhibition of immune effectors is released, and these cells regain their ability to become activated and kill tumour cells. The binding of the PD-1 receptor expressed at the surface of T cells with its cognate ligands, PD-L1 and PD-L2, results in the inhibition of T-cell effector function and decreased cytotoxic activity within the tumor bed. This is consistent with the notion that antibodies targeting the PD-1/PD-L1 axis require the presence of tumor-specific T lymphocytes to be effective. On the other hand, the ubiquitous CTLA-4 has non-overlapping suppressive effects on antitumor immunity, being preferentially involved in controlling the earlier phases of the immune response (priming), primarily in lymphoid organs. These effects occurring at different sites and during different phases of the immune response support the rationale to combine the CTLA-4 blockade with antibodies targeting the PD-1/PD-L1 axis.

With regard to GC, data collected so far indicate that PD-L1 is expressed in about 65% of GC tissues and CTLA-4 is expressed in 86% of cases, whereas these molecules are undetectable in normal gastric mucosa of healthy individuals [74,75,76]. Notably, positive tumour cell staining for PD-L1 or CTLA-4 has been associated with an inferior OS in GC patients and TILs express PD-1, PD-L1, and CTLA-4 molecules at a significantly higher level compared to the T cells of the peripheral blood [77]. A recent meta-analysis carried out on 15 studies, including 3291 GC patients, confirmed that the expression level of PD-L1 in tumour cells significantly correlates with a worse OS. In addition, a subgroup analysis showed that GC patients with deeper tumor infiltration, positive lymph node metastasis, positive venous invasion, Epstein–Barr virus (EBV) infection, or GC showing microsatellite instability (MSI) are more likely to express PD-L1. These findings suggest that GC patients, specifically those with EBV+ and MSI tumors, may be preferred candidates for PD-1-targeting therapies [78]. A FISH analysis demonstrated amplification of the gene encoding for PD-L1 in 11% of EBV+ cases, suggesting that this genetic change may be associated with, or even promote, the clonal evolution and malignant progression of EBV and GC [79]. The expression of PD-L1 by T/NK lymphocytes infiltrating GC may be also of potential prognostic relevance. Functional studies carried out in vitro revealed that blocking PD-1/PD-L1 signalling markedly enhanced cytokine production and cytotoxic activity while inhibiting NK cell apoptosis. Intriguingly, treatment with a PD-1 blocking antibody significantly inhibited the growth of xenografts in nude mice, an effect that was completely abrogated by NK depletion [80].

Like the alternative immune checkpoint molecule, VISTA appears particularly attractive as a potential therapeutic target. VISTA is a type I membrane protein expressed predominantly in myeloid, granulocytic and T cells. Although the ligands for VISTA are not yet known, available evidence indicates that VISTA may serve both as a ligand (for antigen presenting cells) and as a receptor (for T cells), and that VISTA suppresses T-cell activation, a function that could be independent of PD-1/PD-L1 signalling [81]. An analysis of a cohort of 464 therapy-naive GC samples and 14 corresponding liver metastases disclosed that VISTA expression in tumor cells was detected in 41 GCs (8.8%) and two corresponding liver metastases (14.3%), but no significant correlation with patient outcome was observed [82].

TIM-3 is a member of the TNF family and a negative regulator of CD4^+^ helper 1 and CD8^+^ cytotoxic T cells. [83]. It has been reported that the expression of TIM-3 defines a subpopulation of specific PD-1^+^ exhausted CD8^+^ T cells with a low production of IFN-γ, TNF-α and IL-2, thus providing a rationale for combining immunotherapy targeting both TIM-3 and PD-1 inhibitory molecules [84,85].

The anti-CTLA-4 ipilimumab antibody and the anti-PD-1 antibodies, pembrolizumab and nivolumab, were first approved by the US Food and Drug Administration (FDA) for the treatment of patients with metastatic melanomas in 2011 and 2014, respectively. However, data accumulated so far indicates that while anti CTLA-4 antibodies yielded only partially satisfactory results, PD-1/PD-L1 inhibitors show more promising results (Table 4). Interestingly, patients with a post-treatment CEA antigen proliferative response had a median survival time of 17.1 months compared with 4.7 months for non-responders to the anti-CTLA-4, tremelimumab (*p* = 0.004), suggesting a rationale for combinations of CTLA-4 blockade with vaccines targeting GC antigens in the future [86]. Moreover, the efficacy of immunotherapies targeting the PD-1/PD-L1 in different solid tumours stimulated the activation of combination studies with other active targeted biologic agents or immune modulating treatments. Indeed, several clinical trials using new antibodies targeting the PD-1/PD-L1 axis in combination with other immunotherapies are ongoing (Table 4). The rationale supporting the combination of different immunotherapeutic agents is supported by several pre-clinical data which indicate that targeting only one of the complex steps required for the generation of effective anti-tumor immune responses is often insufficient. Moreover, taking into account the ability of several chemotherapeutic drugs to induce immunogenic cell death, therapeutic approaches combining immunotherapy and chemotherapy are also being actively investigated (Table 4).

Combination therapies with immune checkpoint inhibitors have also targeted the subset of HER2-overexpressing tumors which almost invariably become resistant to trastuzumab-containing regimens and progress. Pre-clinical evidence supports the rationale for combining trastuzumab and inhibitors of the PD-1/PD-L1 axis. In fact, it has been demonstrated that HER-2 inhibition can promote T-cell activation and trafficking, enhance IFNγ production by NK cells and boost antibody-dependent cellular cytotoxicity which may efficiently synergize with inhibition of the PD-1/PD-L1 pathway [87]. A phase Ib/II, open-label, dose-escalation study is investigating the novel anti-HER2 mAb, margetuximab, in combination with pembrolizumab in patients with advanced HER2-amplified GC who are refractory to standard trastuzumab-based combination chemotherapy (NCT02689284) [88]. A variety of other combinations is being investigated in which, on the backbone of inhibitors of the PD-1/PD-L1 axis, other drugs target additional nodes in the cancer immunity cycle [89]. The latter include agents inhibiting other immune checkpoints (TIM3, LAG3), T-cell costimulatory agonist antibodies (GITR, OX40, 4-1BB), enzymatic inhibitors (IDO-1), as well as radiation and other cytotoxic drugs. In addition, the combination of nivolumab and GS-5745, a matrix metalloproteinase 9 inhibitor, is also being investigated in patients with unresectable or recurrent GC/GEJ adenocarcinoma (NCT02864381). Combination with radiotherapy, although still poorly explored in the setting of GC, represents another promising therapeutic opportunity. Indeed, single dose and fractionated radiotherapy has been found to upregulate tumor PD-L1 expression in various pre-clinical models but also promotes the immunogenicity of tumor cells through the generation of new antigens or enhanced exposure or release of existing tumor antigens. Therefore, concomitant treatment with anti-PD1 antibodies may overcome the immune suppression activity mediated by PD-L1 that is up-regulated by radiotherapy, thus allowing for the generation of more effective anti-tumor immune responses that may lead to long-term tumor control [90]. Clinical trials involving GC patients are ongoing, including studies combining pembrolizumab with palliative radiotherapy in the metastatic setting, as well as with neoadjuvant chemoradiotherapy for GEJ and gastric cardia cancers in earlier stage resettable disease (NCT02730546) [91].

### 3.4. Agonistic Antibodies for Costimulatory Receptors

The generation of therapeutically effective immune responses requires not only relieving the inhibition of negative regulatory pathways but also promoting T cell activation. T cell costimulation through receptors, like OX40, 4-1BB or ICOS, provides a potent activation signal that actively promotes the expansion and proliferation of killer CD8 and helper CD4 T cells [105,106,107]. Studies carried out in pre-clinical models have demonstrated that treatment with OX40 agonists, including both anti-OX40 mAb and OX40L-Fc fusion proteins, results in tumor regression [105]. These effects are mainly due to the ability of OX40 ligands to promote the survival and expansion of CD8 and conventional, non-regulatory CD4 T cells. On the other hand, it is still unclear whether OX40 activation promotes or inhibits Treg cell responses, as available data in this respect are not univocal [105]. A murine IgG monoclonal agonistic antibody against OX40 was investigated in a phase I clinical trial in 30 patients with metastatic solid malignancies. The treatment was overall tolerable, and six patients achieved stable disease, whereas no partial response was observed [108]. Several phase I clinical trials are currently ongoing with agonistic monoclonal antibodies targeting OX40 as a single therapy or in combination with checkpoint inhibitors [105].

4-1BB (CD137) is an inducible costimulatory receptor expressed by T cells, NK cells, and antigen presenting cells. Activation of 4-1BB by its ligand stimulates the proliferation and activation of T and NK cells [106] Considering that activation of NK cells results in enhanced antibody-dependent cell-mediated cytoxicity (ADCC), treatment with anti-41BB agonists not only increases immune-mediated antitumor activity but may also enhance the therapeutic efficacy of monoclonal antibodies, such as rituximab and trastuzumab, that function through ADCC mechanisms [109]. Gonistic 4-1BB antibodies have demonstrated potent anti-cancer efficacy in murine models and, on the basis of promising pre-clinical findings [110], several clinical trials have been initiated using the utomilumab and urelumab antibodies, mainly in patients with advanced solid tumors.

Inducible costimulator (ICOS) is a T cell costimulatory molecule belonging to the CD28/CTLA-4 family, which promotes the proliferation and cytokine production, mainly of CD4 T lymphocytes [111]. Up-regulation of ICOS is frequently found in activated T lymphocytes, particularly in patients treated with anti-CTLA4 antibodies, and its expression is regarded as a biomarker that is indicative of the binding of an anti-CTLA4 antibody to its cognate target [112]. Notably, the combination of ICOS agonist antibodies with CTLA4 blockade results in strong synergistic effects due to the marked up-regulation of ICOS expression of ICOS after anti-CTLA4 therapy [111]. JTX-2011, GSK3359609 and MEDI-570 are ICOS agonistic monoclonal antibodies that are currently being investigated in phase I/II clinical trials as monotherapies or in combination with checkpoint inhibitors, mainly in patients with advanced solid malignancies.

### 3.5. Safety Issues Related to the Use of Checkpoint Inhibitors

Overall, checkpoint inhibitors are generally better tolerated than chemotherapy regimens administered to patients with GC. Generally, the profiles of side effects that occur with different anti–PD-1/PD-L1 inhibitors are broadly similar [113]. About 10–20% of GC patients treated with anti-PD-1/PD-L1 monotherapy have adverse grade ≥3 events, including fatigue, anemia, and elevated alanine and aspartate aminotransferase levels. Checkpoint inhibitor therapy may also induce immune-related AEs (irAEs) that may affect rheumatic, gastrointestinal, skin, pulmonary, endocrine, neurological, hepatic, cardiac, and renal tissues [114]. In patients with GC, pneumonitis and colitis are the most common grade ≥3 irAEs. Usually, higher rates of treatment-related adverse events are observed in patients treated with anti–CTLA-4 antibodies and combination regimens as compared with anti-PD-1/PD-L1 monotherapies [114]. Although these adverse events are clinically manageable in most cases, long-term sequelae and deaths have been reported in a small proportion of patients [114], pointing to the need to adequately educate healthcare professionals and patients, perform close monitoring, and activate multidisciplinary collaborations to effectively manage these adverse events.

### 3.6. Cancer Vaccines

The therapeutic potential of cancer vaccines is due to their ability to activate and boost anti-tumor immune responses. Dendritic cells (DCs), the critical target of all cancer vaccines, are professional antigen presenting cells that play a pivotal role in orchestrating and coordinating anti-tumor immune responses, and are able to activate NK cells, B lymphocytes, and naïve and memory T cells by presenting tumor antigen/MHC complexes. In GC patients, higher numbers of DCs infiltrating the tumor have been associated with lower lymph node metastases and better patient survival [115]. Several strategies have been used to load DCs with tumor antigen as a vaccine, such as (i) synthetic peptide pulsed on DCs, (ii) DCs engineered with plasmid DNA, RNA, or viruses, (iii) tumor cell lysate (e.g. RNA, whole cell, phagosomes) mixed with immature DCs, (iv) DCs fused with whole tumor cells via PEG or electroporation. The most widely used vaccines are based on DCs pulsed with MHC-restricted peptides derived from known tumor-associated antigens, although the use of DCs in the clinical setting is limited by the short life span of these cells in vivo. The tumor-associated antigens targeted so far by vaccines for GC patients are melanoma-associated antigen (MAGE) A3 [116,117], HER2(_p369_) peptide [116], gastrin-17 diphtheria toxoid (G17DT) [118,119], URLC10 or VEGFR1 epitopes [120] and heat shock protein gp96 [121]; adjuvant BCG (Bacillus Calmette–Guérin) was also tested with chemotherapy [122] (Table 5). To personalize the choice of peptides to be used as vaccines in individual GC patients, pre-vaccination peripheral blood mononuclear cells of each patients were tested for their reactivity in vitro to the repertoire of each MHC peptide, and only the reactive peptides were administered in vivo [120]. Delayed-type hypersensitivity (DTH) to the vaccinated peptides was observed in some patients, whereas increased cellular and humoral immune responses to the vaccinated peptides were observed in others, with a concomitant prolonged survival [123]. Recently, encouraging clinical results were obtained using HLA-A24-restricted vascular endothelial growth factor receptor 1 (VEGFR1)-1084 and VEGFR2-169 peptides, combined with S-1 and cisplatin chemotherapy [120]. Most patients (82%) showed the induction of VEGFR1-specific cytotoxic T lymphocyte responses, twelve patients (55%) showed partial responses and 10 had stable disease after two cycles of the therapy. Notably, patients showing VEGFR-specific T-cell responses had a significant higher OS and time to progression (TTP), indicating that cancer vaccination combined with standard chemotherapy warrants further analysis as a promising strategy for the treatment of advanced GC [124]. To enhance GC vaccine efficacy, antigenic formulations targeting multiple antigens are being explored. In this direction, a cocktail vaccine including multiple peptides (DEPDC1, FOXM1, KIF20, URLC10, and VEGFR1) combined with S-1 chemotherapy was administered as a post-operative adjuvant therapy in a series of pathologically stage III advanced GC patients [125,126]. The treatment was well tolerated, and an optimal relative dose was achieved, paving the way for further studies aiming at assessing the efficacy of this therapeutic strategy. An alternative approach to target multiple antigens is the fusion of DCs with whole tumor gastric cells to generate DC-tumor hybrids, e.g., by the electrofusion technique. These hybrid cells have the advantage of combining the potent antigen presenting capacity of DCs with the availability of the full repertoire of antigens expressed by tumor cells [127,128]. To circumvent the disadvantage of the limited availability of viable autologous tumor cells for the fusion, allogeneic GC cells may be used instead of autologous GC cells (cross-priming antigens) Therefore, it is not necessary to match the HLA haplotype between patients and allogeneic tumor cells used to generate the fusion. Although DC-tumor hybrids are safe and have induced efficient antitumor immune responses in early clinical trials, limited positive clinical responses have been reported in GC, with better results occurring with the use of costimulation with IL-12 [129] and the use of the of combination of TLR2 and TLR4 agonists [130]. 

## 4. Concluding Remarks and Future Perspectives

Over the last decade, our understanding of the mechanisms underlying immune modulation has greatly improved, allowing for the development of multiple therapeutic approaches that are revolutionizing the treatment of cancer. Immunotherapy for GC is still in the early phase but is rapidly evolving. The challenges moving forward are to put much effort into biologic and immunologic exploration in GC setting to fine-tune and tailor, more precisely, the various available or emerging immunotherapeutic approaches. In the near future, it will be necessary to design large prospective trials to validate reliable predictive factors, allowing for the selection of GC patients with the highest chance of benefitting from immunotherapy.

## Figures and Tables

**Table 1:** Tumor stage and associated survival rate.

Tumor Stage	TNM Classification	Survival Rate (%, 5 Years)	Treatment
1	T1-2, N0-1, M0	69	Surgical resection
2	T1-4a, N0-3a, M0	43	Preoperative chemotherapy and surgery followed by post-operative adjuvant chemo/radio-therapy
3	T1-4b, N1-3b, M0	28	
4	Tx, Nx, M0	9	Palliative chemotherapy ± targeted therapy

*TNM Classification of malignant tumors* [2]. T: size of the primary tumor; N: lymph node involvement; M: metastasis.

**Table 2:** Adoptive cell immunotherapy for gastric carcinoma (GC).

Type of Treatment	Setting	Primary End-Point	References
Autologous tumor infiltrating lymphocytes (TILs) combined with rIL-2	advanced GC (*n* = 23)	13% CR 21.7% PR	[57]
Autologous peripheral blood lymphocytes activated by anti-CD3 antibody and interleukin (IL)-2 + chemotherapy	GC with a life expectancy >12 weeks (*n* = 84)	OS in patients that had received surgery was prolonged after EAAL immunotherapy	[58]
*Ex vivo* expanded natural killer (NK) in co-culture with K562			[43]
NK expansion using recombinant human fibronectin fragment (FN-CH296) + target-based chemotherapy	unresectable, locally advanced, and/or metastatic GC (*n* = 3)	phase I trial, good tolerability	[44]
Expanded NK with OK432, IL-2, and modified FN-CH296	unresectable, locally advanced and/or metastatic GC (*n* = 3)	phase I well tolerated with no severe adverse events	[45]
NK-92 cell line	advanced solid tumors	only pre-clinical studies	[29]
Autologous cytokine-induced killer cells (CIK)	post-operative locally advanced GC (*n* = 151)	5-year OS 46.8 vs. 31.4% intestinal type (*p* = 0.045), 5-year DFS 28.3 versus 10.4% (*p* = 0.044)	[59]
Autologous CIK + chemotherapy	post-operative locally advanced GC (*n* = 95)	DFS and OS were longer in pts with higher major histocompatibility complex (MHC)-I-related gene A (MICA)	[58]
Autologous CIK + chemotherapy	post-operative locally advanced GC (*n* = 156)	longer OS	[60]
Autologous CIK + chemotherapy	GC stage II-III (*n* = 226)	longer DFS and OS	[61]
Autologous CIK + oxaliplatin	post-operative stage II-III GC (*n* = 167)	higher 5-year OS rate (56.6% vs. 26.8%, *p* = 0.014) and progression-free survival (PFS) rate (49.1% vs. 24.1%, *p* = 0.026)	[62]
Autologous CIK + FolFox4	post-operative GC (*n* = 51)	reduced GC recurrence rates and enhanced survival rates	[63]

EAAL: expanded activated autologous lymphocytes; DFS: Disease-free survival.

**Table 3:** Engineered adoptive T/NK cells—CAR-T cells.

Type of Treatment	Setting	Type of Study/Trial	Reference/Trial No.
CAR T cell therapy targeting human epidermal growth factor receptor 2 (HER2)	HER2+ GC	pre-clinical studies	[67,69]
CAR-T-like T cells targeting HER2	HER2+ GC	pre-clinical study	[68]
CAR targeting HER2+	HER2-positive solid tumors (breast cancer, ovarian cancer, lung cancer, GC, colorectal cancer, glioma, pancreatic cancer)	ongoing phase I studies	NCT02713984
CAR targeting the carcinoembryonic antigen (CEA)	GC CEA-positive	ongoing phase I studies	NCT02349724 NCT02850536 NCT02416466
CAR targeting Human Mucin-1 (MUC1)	GC MUC1-positive	ongoing phase I	NCT02617134
CAR targeting the epithelial cell adhesion molecule (EpCAM)	GC EpCAM-positive	ongoing phase I studies	NCT02725125 NCT03013712

**Table 4:** Immune checkpoint inhibitors.

Type of Treatment	Setting	Primary End-Point	Reference/Trial No.
Tremelimumab (IgG2 anti B7 ligand of CTLA-4)	metastatic gastric and esophageal carcinomas (*n* = 18)	phase II, OS similar to conventional therapy	[86]
Tremelimumab + Durvalumab	GC/gastroesophageal junction (GEJ) (*n* = 135)	phase Ib/II, ongoing	NCT02340975
Ipilimumab (IgG1κ anti CTL-4)	unresectable locally advanced/metastatic GC/ GEJ (*n* = 143)	phase II, OS similar to conventional therapy	[92]
Ipilimumab + Nivolumab (Anti-PD-1)	GC/GEJ pre-operative setting and nivolumab combined with chemo-radiation	phase Ib, ongoing	NCT03044613
Pembrolizumab (IgG4 anti PD-1)	recurrent or metastatic GC/GEJ (*n* = 39)	phase Ib, 22% partial response, toxicity manageable	[93]
	PD-L1^+^ advanced solid tumors including GC/ GEJ (*n* = 23)	phase Ib, 30% Overall response rate (ORR), median 15 months, better response in patients with high interferon (IFN)-γ gene signature	[94]
	recurrent or metastatic GC/GEJ, 2 line (*n* = 259)	phase II. improved ORR (12%), progression-free survival (PFS) 2 months, and OS 6 months	[95]
	recurrent or metastatic GC/GEJ ≥1% PD-L1+, 1 line	phase II. improved ORR (26%), PFS 3 months, and OS not reach in GC with ≥1% expression of PD-L1	[95]
Pembrolizumab + chemotherapy	recurrent or metastatic GC/GEJ	phase II. improved ORR (60%), PFS 7 months, and OS 14 months	[95]
	recurrent or metastatic GC/GEJ	phase III ongoing	[96]
Pembrolizumab + Ramucirumab (anti VEGFR2)	locally advanced and unresectable or metastatic GC and other tumors (*n* = 155)	phase I, study ongoing	[97]
Pembrolizumab + Margetuximab (anti HER2)	advanced and metastatic GC/GEJ HER2+ (*n* = 72)	phase I, dose escalation, safety, efficacy. Study ongoing	[88]
neoadjuvant Pembrolizumab + chemo/radiotherapy	resectable, locally advanced GEJ or GC of cardia (*n* = 68)	phase Ib/II, side effects and best way to give the treatment. Study ongoing	[91]
Nivolumab (IgG4 anti PD-1)	recurrent or metastatic GC/GEJ (*n* = 160)	phase I/II, ORR 24% Nivolumab and Ipilimumab vs 12% Nivolumab in monotherapy with lower toxicity	[98]
Nivolumab + Ipilumumab	unresectable advanced or recurrent gastric or GEJ cancer, refractory to, or intolerant of, two or more prior chemotherapy regimens, only patients from Asian countries	phase III, improved OS (26.6% at 1 year, median 5.32 months), PFS (1.61 months). ORR 11.2%	[99]
Avelumab (IgG1 anti PD-L1)	advanced or metastatic previously treated solid tumors, including GC/GEJ	phase Ia, dose escalation trial, acceptable toxicity	[100]
	3 line recurrent or metastatic GC/GEJ (*n* = 371)	phase III, Avelumab + best supportive care (BSC) vs BSC ± chemotherapy, study on going at the moment, it did not improve overall survival (OS)	[101]
	unresectable, locally advanced or metastatic GC	Avelumab vs continuation of first-line chemotherapy	[102]
Durvalumab (IgG1κ anti PD-L1)	2/3 line metastatic GC	phase Ib/II Durvalumab or Durvalumab + Tremelimumab vs Tremelimumab alone. study is ongoing	[103]
Durvalumab + Ramucirumab (anti VEGFR2)	refractory GC/GEJ (*n* = 114)	phase Ia/Ib. Safety and efficacy	[104]
Durvalumab + Indoleamine 2,3-dioxygenase (IDO) Inhibitor	selected advanced solid tumors (*n* = 192)	phase I/II safety, tolerability, and efficacy. study ongoing	NCT02318277
Atezolizumab (IgG1κ anti PD-L1)	locally advanced or metastatic solid tumors including GC (*n* = 661)	phase I. Dose escalation Study of the safety and pharmacokinetics. Study is ongoing	NCT01375842
Atezolizumab + IDO inhibitor	locally advanced, recurrent, or metastatic incurable solid tumors including GC (*n* = 158)	phase I. Dose limiting toxicity, adverse events. study is ongoing	NCT02471846
Atezolizumab + FLOT (docetaxel, oxaliplatin, and fluorouracil /leucovorin) chemotherapy	locally advanced unresectable or metastatic GC/GEJ (*n* = 357)	phase Ib/II	NCT03281369
Atezolizumab + Ramucirumab + chemotherapy	GC/GEJ (*n* = 295)	phase II, Atezolizumab + FLOT vs. FLOT. study is ongoing	NCT03421288

**Table 5:** Vaccines.

Type of Vaccine	Setting	Primary End-Point	Reference
DC pulsed with melanoma-associated antigen (MAGE) A3 peptides	MAGE-3-expressing advanced GC (*n* = 12)	phase I, safe and exhibits antitumor effects in some patients	[117]
HER2(_p369_) peptide	advanced or recurrent GC HER2+ (*n* = 9)	phase I, tumor specific T-cell response	[116]
BCG (Bacillus Calmette–Guérin) + chemotherapy	radically resected stage III/IV GC	prolonged 10-year OS (47.1%) as compared to mono-chemotherapy (30%) or surgery alone (15.2%)	[122]
gastrin-17 diphtheria toxoid (G17DT) + chemotherapy	metastatic GC/GEJ (*n* = 94)	phase II, longer TTP and OS in responders	[118]
URLC10 or VEGFR1 Epitopes	chemotherapy-resistant advanced GC (*n* = 14)	phase I, tumor specific T cell responses	[120,124]
heat shock protein GP96 + oxaliplatinum	GC (*n* = 45)	phase II, 81.9% 2-year OS	[121]
OTSGC-A24 (5 HLA-A24-restricted peptides DEPDC1, FOXM1, KIF20, URLC10, and VEGFR1)	inoperable/unresectable, metastatic GC, 2 line therapy or greater (*n* = 23)	favourable results for safety and immune reactivity	[126]
